# Change in gait speed and fall risk among community-dwelling older adults with and without mild cognitive impairment: a retrospective cohort analysis

**DOI:** 10.1186/s12877-023-03890-6

**Published:** 2023-05-25

**Authors:** Claire E. Adam, Annette L. Fitzpatrick, Cindy S. Leary, Anjum Hajat, Sindana D. Ilango, Christina Park, Elizabeth A. Phelan, Erin O. Semmens

**Affiliations:** 1 School of Public and Community Health Sciences, University of Montana, Missoula, USA; 2 Center for Population Health Research, University of Montana, Missoula, USA; 3 Department of Family Medicine, University of Washington, Seattle, USA; 4 Department of Epidemiology, School of Public Health, University of Washington, Seattle, USA; 5 Department of Global Health, University of Washington, Seattle, USA; 6 Division of Gerontology and Geriatric Medicine, University of Washington, Seattle, USA; 7 Department of Health Systems and Population Health, University of Washington, Seattle, USA

**Keywords:** Falls, Change in gait speed, Mild cognitive impairment

## Abstract

**Background:**

Although slow gait speed is an established risk factor for falls, few studies have evaluated change in gait speed as a predictor of falls or considered variability in effects by cognitive status. Change in gait speed may be a more useful metric because of its potential to identify decline in function. In addition, older adults with mild cognitive impairment are at an elevated risk of falls. The purpose of this research was to quantify the association between 12-month change in gait speed and falls in the subsequent 6 months among older adults with and without mild cognitive impairment.

**Methods:**

Falls were self-reported every six months, and gait speed was ascertained annually among 2,776 participants in the Ginkgo Evaluation of Memory Study (2000–2008). Adjusted Cox proportional hazards models were used to estimate hazard ratios (HR) and 95% confidence intervals (CI) for fall risk relative to a 12-month change in gait speed.

**Results:**

Slowing gait speed over 12 months was associated with increased risk of one or more falls (HR:1.13; 95% CI: 1.02 to 1.25) and multiple falls (HR:1.44; 95% CI: 1.18 to 1.75). Quickening gait speed was not associated with risk of one or more falls (HR 0.97; 95% CI: 0.87 to 1.08) or multiple falls (HR 1.04; 95% CI: 0.84 to 1.28), relative to those with a less than 0.10 m/s change in gait speed. Associations did not vary by cognitive status (p_interaction_ = 0.95 all falls, 0.25 multiple falls).

**Conclusions:**

Decline in gait speed over 12 months is associated with an increased likelihood of falls among community-dwelling older adults, regardless of cognitive status. Routine checks of gait speed at outpatient visits may be warranted as a means to focus fall risk reduction efforts.

**Supplementary Information:**

The online version contains supplementary material available at 10.1186/s12877-023-03890-6.

## Background

Falls in older adults are prevalent and affect health at both the population and individual level [1, 2]. Annually, 20–33% of adults 65 and over fall [1, 3] resulting in death, injury, and decreased independence [1, 3, 4] and it is critically important to identify those at risk of falling as early as possible so that prevention strategies can be implemented. Gait speed has been identified as a predictor of fall risk, but most research assessing the relationship between gait speed and falls has focused on gait speed measured at a single time point, using either a gait speed cut-off, (frequently 1 m/second (m/s)) [5–7], or gait speed as a continuous variable [8]. Cross-sectional gait speed measurements are considered to have low predictive value for fall risk overall [9] and specifically for those without a previous history of falls [10].

By contrast, change in gait speed has the potential to be a useful measure of fall risk. Declines in gait speed have been found to be associated with an increased risk of multiple adverse health outcomes (e.g., disability) [11–13] and may also be associated with increased fall risk [14–16]. If confirmed, change in gait speed could lead to earlier identification of older adults at heightened fall risk while they are on a trajectory of gait speed decline [15]. An individual with a starting gait speed of 1.5 m/s, would experience a 33% decrease in gait speed before reaching 1.0 m/s, a commonly applied threshold used to identify individuals as having a higher fall risk. Additionally, change in gait speed could be used to identify older adults whose gait speed is below the commonly applied fall risk threshold (i.e., 1.0 m/s), but who are experiencing a further decline in gait speed. Change in gait speed and fall risk has been investigated previously, but those studies were limited by small sample sizes [17, 18], short study duration, and infrequent measures of gait speed [14, 19]. While gait speed typically declines with advancing age [20], increases in gait speed do occur in older adults, for example following participation in balance and exercise programs [21–24], which is in turn associated with decreased fall risk [24]. Improvements in gait speed are often an outcome measure for fall prevention programs [22, 23].

Mild cognitive impairment (MCI) is common in older adults and the prevalence increases with age (15% ages 75–79, 25% 80–84) [25]. Older adults with MCI experience an even greater fall risk [26] but are frequently excluded from studies investigating fall risk. There is a need for more screening tools for fall risk for older adults with cognitive impairment [27]. Slow gait speed has an established association with increased fall risk for people with intact cognition, and in a limited number of studies, for people with cognitive impairment [6, 8, 28–30]. We accessed data from a longitudinal study that included a large number of older adults both with and without mild cognitive impairment who were followed over several years with frequent measures of gait speed and falls. This study presented the opportunity to quantify the association between change in gait speed and fall risk in older adults, and to determine if this association differed by cognitive status. Previous studies have found that older adults with MCI and without slow gait speed, have an increased fall risk in comparison to older adults with intact cognition and without slow gait speed [6, 31]. These studies provide evidence of an increased baseline risk of falling for people with MCI, independent of gait speed [6, 31]. While there is evidence that fall risk for older adults with MCI and slow gait speed is higher than for older adults without MCI and slow gait speed, there is uncertainty about whether the increased risk from slow gait speed is equivalent in older adults with and without MCI [6]. Given that MCI is a strong independent predictor of falls [26], the contribution of change in gait speed to fall risk among those with MCI may be less compared to those with intact cognition [6]. Our hypothesis was that a decline in gait speed would be associated with increased fall risk in older adults relative to no change in gait speed, and that this relationship would be stronger (e.g. a greater increase in risk) for older adults with intact cognition compared to those with mild cognitive impairment, given that MCI is a strong independent predictor of falls [26].

## Methods

This study utilized data from the Ginkgo Evaluation of Memory study (GEMS), initiated in 2000 and completed in 2008. At baseline, GEMS included 3,069 adults 75 years and older, from four different locations (Winston-Salem, NC; Hagerstown, MD, Sacramento, CA; and Pittsburgh, PA) in the United States [32]. Participants were all community-dwelling and free of dementia at baseline although those with mild cognitive impairment (MCI) were allowed to enroll. GEMS was a randomized controlled clinical trial which allocated participants to 240 mg of Ginkgo biloba per day or placebo [32]. Results of the trial were negative but this cohort has provided extensive data for use in evaluating new questions related to aging [33]. After the baseline and screening visits, study visits were every six months for up to 16 total study visits (eight years of follow up). There have been multiple publications from GEMS where further details on study design and methods can be found [32–34]. GEMS received institutional review board (IRB) approval from all study sites. The study reported here was approved by the University of Montana IRB.

### Participants

**Fig. 1:**
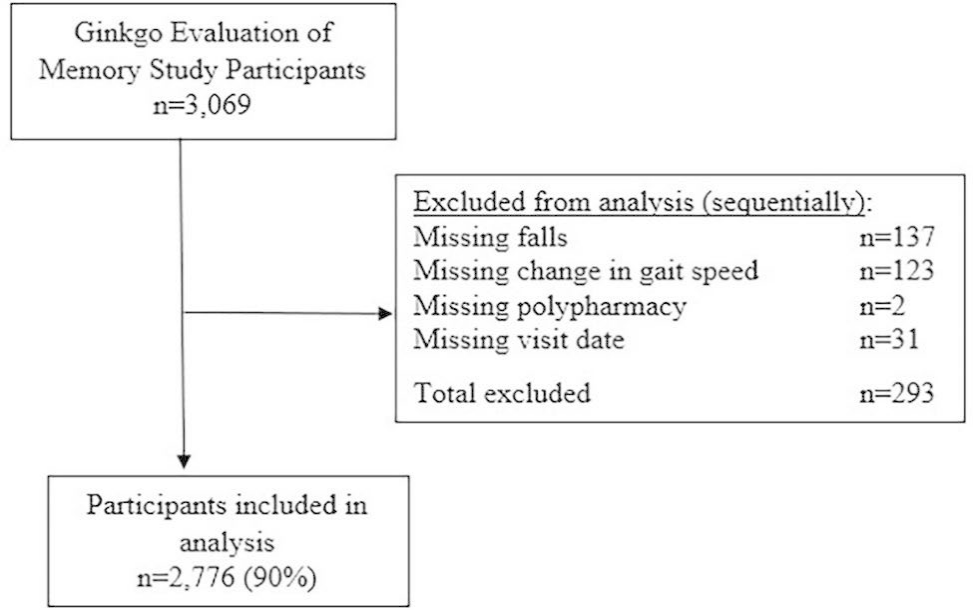
Participants excluded from analyses, by reason for exclusion

Of the original 3,069 GEMS participants, 2,776 participants (90.4%) (Fig. 1.) were included in these analyses with a total of 10,639 observations (median = 3). Participants were excluded from analyses sequentially, starting with missing data for falls (n = 137), then change in gait speed/missing gait speed measurements (n = 123), polypharmacy (n = 2), and for visit dates (n = 31) (Fig. 1).

### Outcome measure

**Fig. 2:**
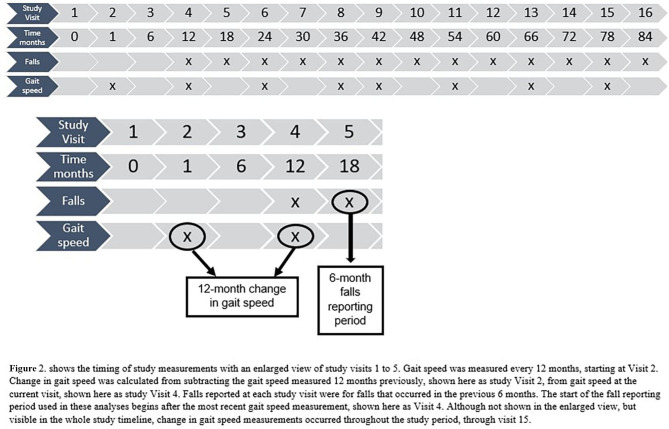
Timing of gait speed and fall measurements in GEMS

Falls were reported at each six-month study visit, beginning at the one-year study visit. Participants were asked if they had experienced any falls in the previous six months and could respond “yes,” “no”, or “don’t know.” (Fig. 2). Responses from participants who answered “don’t know” were coded as missing (less than 1% of observations for falls). If participants reported falling, they were also asked about the number of falls they had. The fall outcomes were no falls vs. all falls (one or more falls), and one fall or no falls vs. multiple falls (two or more falls), reported in the six-month period.

### Exposure measure

The exposure of interest in this study was 12-month change in gait speed. Gait speed was measured beginning at the second study visit. Gait speed measurements occurred approximately annually, at every other study visit, as part of the Functional Assessment performed in GEMS (Fig. 2). Gait speed measurements that occurred more frequently than 12 months apart (two study visits in a row) or less frequently than 12 months apart (at least three study visits apart) were excluded from the analysis to ensure that all change in gait speed measurements were 12 months apart. Of the 12,767 gait speed measurements that had a prior gait speed measurement (e.g.: not the first gait speed measurement in the study), 88% (11,264) were two study visits apart (approximately 12 months), 3% (428) were one study visit apart (6 months), and 8% (1075) were more than three study visits apart (approximately 18 months or more). Gait speed measurements that occurred more or less frequently than two study visits apart (n = 1503) were excluded from analyses. The collection period for falls was from zero to six months after the gait speed measurement. Gait speed was measured from a static start over 4.57 m (15 feet) and recorded by GEMS study staff. Participants were given instructions to walk at their usual pace. Time to walk 4.57 m was converted to gait speed in meters per second (m/s). Improbably fast gait speeds, potentially due to measurement or recording errors, were excluded from analyses using a cut-point for gait speed faster than 1.93 m/s for males (n = 25), and faster than 1.80 m/s for females (n = 18) as these values exceeded the mean preferred gait speed plus three standard deviations by gender for adults ages 75–84 [20]. These values were excluded from analyses, however, all these participants had additional in-range measurements that were included in the analyses. Twelve-month change in gait speed was time-varying and aligned with the start of the six-month fall reporting period (Fig. 2). Change in gait speed was categorized based on clinically measurable differences in gait speed [5, 35], change in gait speed used in prior studies [14, 16, 36, 37], and the distribution of change in gait speed among study participants. We categorized change in gait speed into three groups: (1) faster gait speed (e.g., at least 0.10 m/s increase in gait speed over 12-months), (2) no change (e.g., a change of less than 0.10 m/s in gait speed over 12-months), and (3) slower gait speed (e.g., at least 0.10 m/s decline in gait speed over 12-months).

### Covariates

Covariates selected *a priori* included gender, study treatment (yes/no Ginkgo), study site, and cognition. A three-category time-varying covariate for cognition was created: intact cognition, MCI, and dementia. Intact cognition (no MCI or dementia) and MCI were the cognitive categories of interest for this study. Dementia was the outcome of interest for the original GEM study, and participation in the study ended if dementia was diagnosed. Some participants who developed dementia (n = 463, (17%)) had gait speed and falls data from the study visit where they triggered additional screening for dementia. Dementia was included as a level in the categorical cognitive variable to exclude people with dementia from the intact cognition and MCI levels, but not as a primary objective of analysis for the study. Guidelines from the International Working Group on Mild Cognitive Impairment were used for the determination of MCI, and included a Clinical Dementia Rating global score of 0.5 and a score in the 10th percentile or below (compared to normative data from the Cardiovascular Health Study) on at least 2 of 10 neuropsychological tests [34, 38]. MCI was ascertained at baseline, every 6 months for those participants who failed the cognitive screening, and then annually for all participants beginning at study year four. MCI status at baseline was carried forward until the annual assessments began, unless a participant failed the cognitive screening and had a full cognitive assessment before study year four. Participants were screened for dementia at each six-month study visit using the Modified Mini-Mental State Examination, the Clinical Dementia Rating Scale and the Alzheimer Disease Assessment Scale [33]. Participants who failed the screening exam were then given a neuropsychological battery which included 10 cognitive tests. Those test scores were reviewed by an expert panel, followed by a full neurological examination and magnetic resonance imaging (MRI), after which dementia status was ascertained [33]. Participants who did not meet the criteria for MCI or dementia were considered to have intact cognition. Additional covariates were considered for inclusion in the models. History of heart attack, stroke, or cancer at baseline, hospitalization in the previous six months specifically for cardiac disease, peripheral vascular disease, or stroke, polypharmacy in the previous six months, prior gait speed (measured 12 months earlier), any prior falls in the study period, education at baseline, and current assistive device use were all considered and assessed for inclusion in the final models using bivariate analysis. At baseline, participants were asked if they had a history of cancer, heart attack, or stroke. Hospitalization in the previous six months by participant report for four comorbidities; “heart attack, myocardial infarction, angina, or chest pain due to heart disease or any heart surgery”, “heart failure or congestive heart failure”, “peripheral vascular disease, intermittent claudication, or leg pain from blockage of the arteries”, “stroke, cerebral vascular accident, mini-stroke, or a transient ischemic attack” were included in analyses as a dichotomous variable for each of the four reasons for hospitalization. Participants were asked at each study visit about their medication use. Prescription medication use was included as a dichotomous covariate for polypharmacy (taking five or more prescription medications) [39], since polypharmacy is associated with falls [40]. Previous gait speed, 12 months prior to current gait speed, was also included in the model as a continuous time-varying covariate to account for differences in starting gait speed, which is associated with fall risk, and differences in the relative change in gait speed (e.g.: 0.10 m/s is a larger percent change for a participant with a gait speed of 0.8 m/s than for a participant with a gait speed of 1.0 m/s). To further assess for differences in the association between change in gait speed and fall risk based on gait speed, previous gait speed by category (0.8 m/s or less than, + 0.8 to 1.0 m/s, + 1.0 m/s) was used to assess for effect modification. Cumulative previous falls were calculated and categorized as zero falls, one fall, and two or more falls and were included as a time varying covariate in the model and used to assess for effect modification. There is evidence that fall risk increases with each fall an individual experiences [41]. Categories were selected based on distribution in the data set and interpretability. Education was included as a categorical variable: high school graduation or less, some college, college graduate, post-graduate education. Participants were allowed to use an assistive device during the measurement of gait speed, and assistive device was included as a dichotomous variable (yes/no).

### Statistical analysis

### Modeling Approach

Cox proportional hazards models for recurrent events were used for the primary analyses [42–44]. The recurrent events models allowed for inclusion of falls reported at multiple visits throughout the study, and multiple measurements of change in gait speed for each participant. Effect modification on the multiplicative scale by cognition was assessed using models with an interaction term for change in gait speed and cognition. The likelihood ratio test was used to compare models with and without interaction terms. Schoenfeld residuals were assessed to check the proportional hazards assumption [42]. All analyses were completed with the statistical software R.

### Sensitivity analyses

Interaction with previous gait speed and previous number of falls.

We assessed effect modification on the multiplicative scale by previous gait speed and previous number of falls using an interaction term for change in gait speed and previous gait speed, and change in gait speed and previous number of falls.

### Imputation

For some visits, there were measurements of the exposure and outcome, but the visit date was missing (204 (1.9%) observations). When visit date was missing, age was imputed based on the baseline age and the current visit number, assuming all visits were six months apart. Imputed age was then used when there was missingness for the time axis variables. Missing change in gait speed was imputed in a two-part process [45]. First, if available, the reason for missingness was used to impute gait speed. For the following reasons, gait speed was recorded as 0.01 m/s for: “tried but unable, you felt it was unsafe, participants felt it was unsafe, participant cannot walk even with support, participant unable to understand instruction.” If the reason was further described, such as an injury, gait speed was recorded as 0.01 m/s, but for reasons such as not enough space, or study staff forgot to take a gait speed measurement, values were imputed using multiple imputation. Updated change in gait speed and previous gait speed values were calculated based on this first round of imputation. For continued missing change in gait speed, previous gait speed, and polypharmacy, a time varying approach of multiple imputation with expectation maximization with bootstrapping was used [46]. Pooled confidence intervals were calculated using Rubin’s Rules [47]. Hazard ratios and 95% confidence intervals from the original analyses were then compared with the results from the analyses with imputed data.

### Competing Risks

GEMS was not originally designed to assess the association between change in gait speed and falls; over time, some participants left the study early if they died, were lost to follow-up, or developed dementia. The purpose of this competing risks analysis was to determine if participants who left the study early had a risk of falling that differed from those participants who remained in the study for longer that altered the association between change in gait speed and falls. Inverse probability weights were created using a Cox model based on the association between covariates used in the full model and risk of leaving the study early due to dementia, death, or loss to follow-up, and then added to the fully adjusted, weighted Cox model for change in gait speed and falls. The unweighted and weighted models were compared to determine if the HR for change in gait speed and falls changed when participants who were more likely to leave the study early were upweighted.

## Results

Participants had a mean age of 78.5 at baseline, were predominantly male (55%), White (96%), and had attended at least some college (mean education = 14.5 years) (Table 1). 14% of participants (n = 377) had MCI and 30% (n = 770) had polypharmacy at the one-year study visit (Table 1). The prevalence of falls during the study period was 48% (n = 1344), and 19% (n = 490) at the one-year study visit (Table 12). Participants who fell during the study period were more likely to be female (47% n = 625), have MCI (23% n = 314) and have slower gait speed (0.86 m/s) than participants who did not fall during the study period (Table 2).The mean gait speed at the one- year study visit was 0.93 m/s (SD = 0.2). Of the 2,088 falls that occurred during the study period, 30% (617) occurred with a prior gait speed of greater than 1.0 m/s.

**Table 1:** Baseline and one-year characteristics of GEMS participants included in analyses (n = 2776)

Characteristic	Mean or N (SD or %)
*Measured at Baseline*	
Age (years)	78.5 (3.2)
Gender- Female	1256 (45%)
Race – White	2650 (96%)
Treatment- Ginkgo	1411 (51%)
Education (years)	14.5 (3.1)
History of Heart Attack^a^	255 (9%)
History of Stroke^a^	73 (3%)
History of Cancer^a^	518 (19%)
*Measured at one-year visit* ^*a*^	
MCI	377 (14%)
Polypharmacy-yes	770 (30%)
Gait speed (m/s)	0.93 (0.20)
Change in gait speed (m/s)	0.014 m/s slower (0.21)
All falls	490 (19%)
Multiple falls	132 (5%)

^a^Missing values: Heart attack [36], stroke [49], cancer [1], one year visit (132).

**Table 2:** Characteristics of participant by cumulative fall status at the last observed visit (n = 2776)

Characteristic	All participants Mean or N (SD or %) n = 2776	No Fall (s) Mean or N (SD or %) n = 1432 (52%)	Fall (s) Mean or N (SD or %) n = 1344 (48%)
Age	83.0 (3.4)	82.5 (3.3)	83.4 (3.4)
Gender (Female)	1256 (45%)	631 (44%)	625 (47%)
MCI	593 (21%)	279 (20%)	314 (23%)
Polypharmacy	1134 (41%)	531 (37%)	603 (45%)
Gait speed (m/s)	0.88 (0.22)	0.90 (0.21)	0.86 (0.23)
Change in gait speed (m/s)- slower	0.03 (0.18)	0.03 (0.18)	0.04 (0.18)

Cox proportional hazards models for recurrent events were used for the analysis of the association between change in gait speed and fall risk. All models were adjusted for gender, study site and treatment (Ginkgo) (Table 3). Each subsequent model included all of the variables from the previous model and an additional variable, for example Model 3 was adjusted for previous gait speed and previous falls (Table 3). Previous gait speed, prior falls, cognition (intact cognition, mild cognitive impairment, and dementia) and polypharmacy were included in the full models. Gender, study site, and polypharmacy were included in the models as stratified variables to account for violations of the proportional hazards assumption [42].

**Table 3:** The association between 12-month change in gait speed and fall risk (N = 2776, observations = 10,639^a^)

Model	HR	95% CI
Model 1		
Reference is no change (0.10 m/s faster to 0.10 m/s slower)		
Faster	0.96	0.86 to 1.07
Slower	1.07	0.97 to 1.17
Model 2 = Model 1 + previous gait speed		
Faster	0.92	0.82 to 1.03
Slower	1.16	1.05 to 1.29
Model 3 = Model 2 + previous falls (categorical)		
Faster	0.96	0.86 to 1.07
Slower	1.14	1.03 to 1.27
Model 4 = Model 3 + cognition		
Faster	0.96	0.86 to 1.07
Slower	1.14	1.03 to 1.26
Model 5 = Model 4 + polypharmacy^b^		
Faster	0.97	0.87 to 1.08
Slower	1.13	1.02 to 1.25

*Note*: All models adjusted for gender^b^, study site^b^, and treatment (Ginkgo), and each model adjusted for all covariates in the previous model and the listed covariate(s). ^a^4919 (46%) of observations are for the reference group, 2531 (24%) are for faster gait speed, and 3189 (30%) are for slower gait speed ^b^Stratified variable. Cognition: (Intact, MCI, dementia)

Decreased gait speed of more than 0.10 m/s was associated with a HR for all falls 1.13 (95% CI: 1.02 to 1.25) and multiple falls 1.44 (95% CI: 1.18 to 1.75) in the next six months in models adjusted for gender, treatment (Ginkgo), study site, previous gait speed, cognition, polypharmacy, and previous number of falls (categorical) with age as the time axis (Tables 3 and 4). Increased gait speed of more than 0.10 m/s was associated with a HR of 0.97 (95% CI: 0.87 to 1.08) for all falls and HR of 1.04 (95% CI: 0.84 to 1.28) for multiple falls in the next 6 months in a fully adjusted model. A decline in gait speed was significantly associated with falls when previous gait speed was adjusted for in the model.

**Table 4:** The association between 12-month change in gait speed and risk of multiple falls (N = 2776, observations = 10,639^a^)

Model	HR	95% CI
Model 1		
Reference is no change (0.10 m/s faster to 0.10 m/s slower)		
Faster	1.03	0.84 to 1.27
Slower	1.21	1.01 to 1.45
Model 2 = Model 1 + previous gait speed		
Faster	0.92	0.75 to 1.14
Slower	1.53	1.26 to 1.86
Model 3 = Model 2 + previous falls (categorical)		
Faster	1.03	0.84 to 1.26
Slower	1.49	1.22 to 1.82
Model 4 = Model 3 + cognition		
Faster	1.04	0.85 to 1.27
Slower	1.47	1.21 to 1.80
Model 5 = Model 4 + polypharmacy^b^		
Faster	1.04	0.84 to 1.28
Slower	1.44	1.18 to 1.75

*Note*: All models adjusted for gender^b^, study site^b^, and treatment (Ginkgo), and each model adjusted for all covariates in the previous model and the listed covariate(s). ^a^4919 (46%) of observations are for the reference group, 2531 (24%) are for faster gait speed, and 3189 (30%) are for slower gait speed. ^b^Stratified variable

**Fig. 3:**
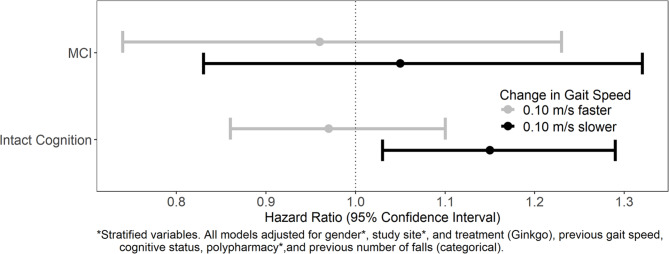
Interactions between change in gait speed and cognition status, for all falls

**Fig. 4:**
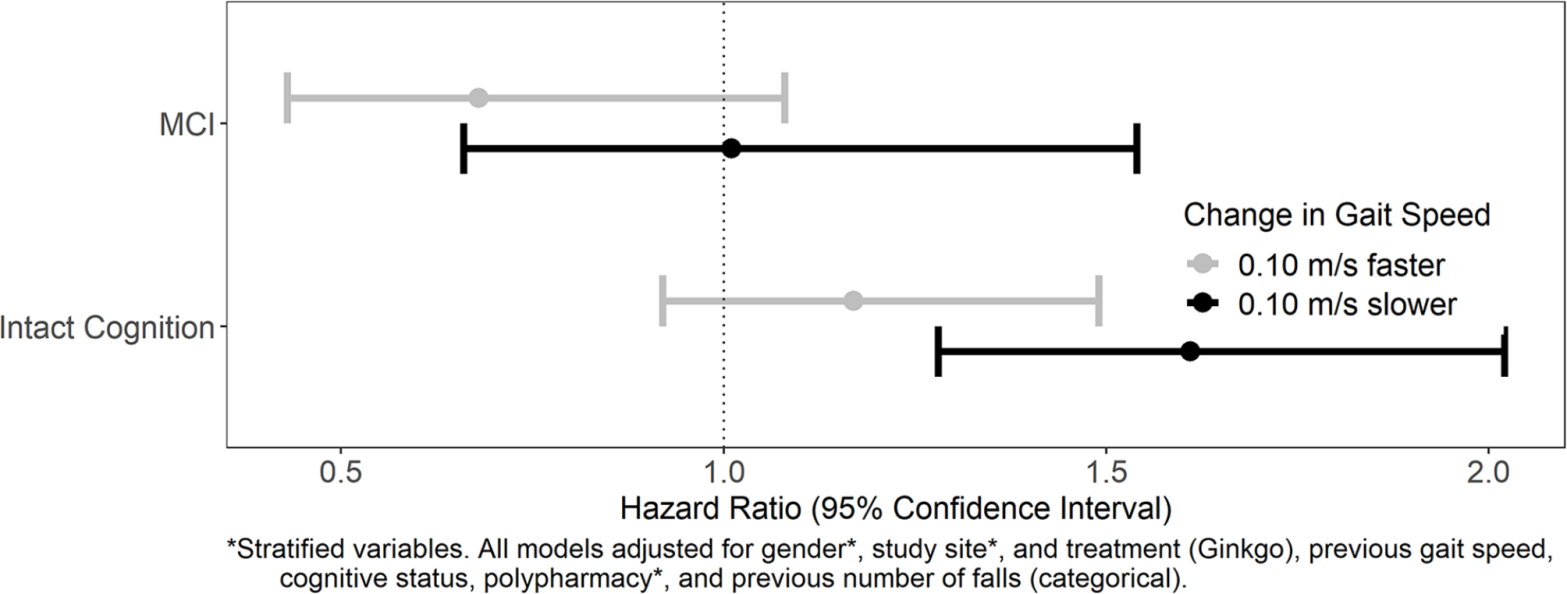
Interactions between change in gait speed and cognition status, for multiple falls

Proportional hazards assumptions were met for all models. The interaction between change in gait speed category and cognition was not statistically significant for all falls or multiple falls (Figs. 3 and 4 and p = 0.95 all falls, 0.25 multiple falls).

### Sensitivity analyses

Interaction with previous gait speed and previous number of falls.

There was no evidence of a significant interaction between change in gait speed category and previous gait speed category (Figures S1 and S2, p = 0.12 all falls, 0.47 multiple falls), or change in gait speed category and previous number of falls category (Figure S3, p = 0.68 all falls).

### Imputation

Imputation increased the number of observations from 10,639 to 14,085 (Table S1). Using the first stage of imputation for gait speed, incorporating reason for missingness, 222 values were imputed for gait speed resulting in 256 additional values for change in gait speed. Any missingness for the time axis, age, was imputed based on age at the second visit and current visit number, assuming all visits were exactly six months apart. In the second stage, using multiple imputation, values were imputed for polypharmacy (< 0.1%), previous fall category (< 0.4%), cognition (< 0.1%), previous gait speed (17%), and change in gait speed (21%). For slower gait speed, for both all falls and multiple falls, multiple imputation attenuated hazard ratios 1.11 to 1.08 (imputed, all falls), 1.44 to 1.34 (imputed, multiple falls)) and expanded confidence intervals (Table S1). For slower gait speed and all falls, the confidence interval expanded to include 1 (0.95 to 1.23). For faster gait speed, the CIs for multiple imputation and all falls expanded, and for multiple falls was the same size. The hazard ratio for all falls changed from 0.97 to 0.96 (imputed) for all falls, and from 1.04 to 1.02 (imputed) for multiple falls. The 95% CIs remained overlapping between all imputed and non-imputed models.

### Competing risks

Inverse probability weights were used for the competing risks analysis to adjust for participants (n = 532 (19%)) who left the study early before visit eight (first interquartile range for maximum visit number, mean maximum visit = 10.7, approximately 48 months). Using the weights for change in gait speed (truncated 1–99%, n = 2755, mean IPW = 1.01) did not change the interpretation of the results; confidence intervals for more rapid change in gait speed for both weighted and unweighted models for all falls and multiple falls included 1, and slower change in gait speed was associated with an increased HR for both weighted and unweighted models for all falls and multiple falls (Table S2). The HR for decreased gait speed was greater for the weighted model than the unweighted model (1.12 vs. 1.11, all falls, 1.42 vs. 1.40 for multiple falls), however the confidence intervals remained overlapping.

## Discussion

We found that a decline in gait speed was associated with fall risk in older adults with and without mild cognitive impairment, and this association is stronger for multiple falls than for all falls. We also found that the gait speed for 30% of observations prior to falls was above the commonly used 1 m/s threshold. These results suggest that change in gait speed may be a useful alternative to a gait speed threshold to more precisely identify older adults at increased risk of falls. This is a potential tool that is inclusive for older adults with differences in cognitive status, gait speed, and prior fall history. Declining gait speed is associated with multiple poor health outcomes and typically results from muscle weakness [48], pain [48], impaired balance [49], impaired cognitive function [12], and underlying comorbidities [5, 29], all of which are also associated with increased fall risk [26, 50, 51], providing the scientific rationale for using gait speed as a screening tool for fall risk.

Our study is unique in comparison to other published studies examining change in gait speed and fall risk due to the inclusion of older adults both with and without mild cognitive impairment who had undergone multiple rigorous cognitive assessments. Understanding the association between gait speed and fall risk in older adults with and without cognitive impairment is critical to assess whether gait speed could be used as a screening tool for fall risk in both populations. In comparison to two previous studies that had a similar timing for change in gait speed (12-month change in gait speed) and magnitude of change in gait speed, our results aligned with one of these that found an association between a decline in gait speed of > 0.15 m/s and increased risk of a fall; however, this study did not specifically include an assessment for mild cognitive impairment [14]. Our results differed from a study which included only people with MCI (n = 110) and found an association between a > 0.1 m/s decrease in gait speed and falls with injuries requiring an emergency room visit, but not all falls [16].

Our study had numerous strengths, including a large number of participants with frequent measures of gait speed and falls over multiple years. The study participants were at high risk for falls based on their age [1]. Therefore, studying fall risk in this population is critical. Data on important confounders such as polypharmacy and hospitalizations were available. Frequent assessment of dementia allowed for identification of participants who had transitioned from intact cognition or MCI to dementia during the study period. We accounted for missingness by conducting two sensitivity analyses: competing risks and imputation. Our competing risks analysis results provided evidence of a stronger relationship between a decline in gait speed and increased fall risk in the weighted model accounting for censoring and loss to follow-up. While the results of the multiple imputation (MI) analysis showed a weaker association between a decline in gait speed and increased fall risk, we have concerns about imputing change in gait speed. In general, multiple imputation decreased the precision of our estimates as evidenced by wider confidence intervals. This is potentially due to a lack of covariates related to both acute changes in gait speed and fall risk, such as injury, pain, and acute illness, and therefore we utilized MI as a sensitivity analysis rather than the primary analysis [48, 50, 52].

While the study had excellent ascertainment of MCI, the timing of the measurement of MCI was a potential limitation of the study. Assessment of MCI changed during the course of the GEM study, in that it was only ascertained for participants who failed screening for dementia during the first four years but thereafter was measured annually. In this first four-year period, participants may have transitioned between normal cognition and MCI without this being captured in the analysis; in a similar study population during an approximately four-year time period, 18-25% of participants transitioned between both intact cognition and MCI [53]. Potential misclassification of cognitive status during the first four years may have contributed to our lack of finding a difference in the strength of association between change in gait speed and fall risk by cognitive status. Additionally, the wide confidence intervals for the interaction between cognitive status and change in gait speed, means that these data should be interpreted with caution. It could be that there is no evidence to support an interaction between change in gait speed and cognitive status, or we are underpowered to detect an effect [54]. Because participants were only asked about falls every six months, and not more frequently, there may have some inaccuracy in the reporting of falls. Some participants responded that they did not know if they had a fall in the previous six months, however, this occurred so infrequently, it is unlikely to have impacted findings. Participants with MCI or dementia may have impaired recall of fall events, which could lead to underestimates of falls among these individuals. Additionally, some falls data were not incorporated into the analyses as they did not align with the measurement of gait speed; however, this was accounted for in the Cox models by including cumulative previous falls as a covariate. The purpose of incorporating cumulative falls into the analysis was to account for the possibility that previous falls could be associated both with increased risk of future falls [41], and decreased gait speed. The participants in this study were predominantly White, highly educated, and community dwelling at baseline, all of which limits the generalizability of these findings.

## Conclusions

Our study provides evidence that a decrease in gait speed is a marker of increased likelihood of falls among older adults, regardless of cognitive status. A gait speed threshold of 1.0 m/s is generally accepted in clinical practice as a marker for increased fall risk [5] (1.0 m/s). In our study, we found that 30% of participants in our study had gait speeds faster than 1.0 m/s prior to falling, suggesting that fall risk increases prior to this threshold. Our results also add to the evidence that declines in gait speed are associated with fall risk and this association did not vary significantly by MCI status. Raising awareness among healthcare providers of the utility of measuring gait speed over time in their older patients is an important next step in the dissemination of our study’s findings. Improving the identification of older adults at increased fall risk is essential for tackling the growing public health challenge of falls [55, 56].

## Electronic supplementary material

Below is the link to the electronic supplementary material.

Table S1. The association between change in gait speed and fall risk using multiple imputation

Table S2. The association between change in gait speed and fall risk, competing risks analysis. (N=2755 observations=10424)

Figure S1. Interactions between change in gait speed and previous gait speed category, for all falls

Figure S2. Interactions between change in gait speed and previous gait speed category, for multiple falls

Figure S3. Interactions between change in gait speed and previous falls category, for falls

## Data Availability

The datasets analysed during the current study are not publicly available due to protecting the privacy of the participants but are available from the University of Washington Collaborative Health Studies Coordinating Center (chsccweb@u.washington.edu) on reasonable request.

## **List of Abbreviations:**

(HR): Hazard Ratio
(CI): Confidence Interval
(MCI): Mild Cognitive Impairment
(m/s): meters per second
(GEMS): Ginkgo Evaluation of Memory study
(IRB): Institutional Review Board
(IPW): Inverse Probability Weights
(MI): Multiple Imputation
