# Supplementary material for: Change in gait speed and fall risk among community-dwelling older adults with and without mild cognitive impairment: a retrospective cohort analysis
Source: BMC Geriatr. 2023 May 25;23:328. doi: 10.1186/s12877-023-03890-6 (PMC10214622; doi:10.1186/s12877-023-03890-6)
Supplement: Supplementary file 1 — Table S1. The association between change in gait speed and fall risk using multiple imputation [file 12877_2023_3890_MOESM1_ESM.docx]

**Table S1. The association between change in gait speed and fall risk using multiple imputation.**

| Model | Observations (N) | HR | 95% CI |
| --- | --- | --- | --- |
| All Falls  Reference is no change (0.10 m/s faster to 0.10 m/s slower)  Faster  Slower | 10639 | 0.97  1.11 | 0.89 to 1.06  1.03 to 1.21 |
| With pooled results from 10 imputations  Faster  Slower | 14085 | 0.96  1.08 | 0.85 to 1.08  0.95 to 1.23 |
| Multiple falls  Reference is no change (0.10 m/s faster to 0.10 m/s slower)  Faster  Slower | 10639 | 1.04  1.44 | 0.84 to 1.28  1.18 to 1.75 |
| With pooled results from 10 imputations  Faster  Slower | 14085 | 1.02  1.34 | 0.83 to 1.27  1.04 to 1.73 |

*Note:* All models adjusted for gender^a^, study site^a^, and treatment (Ginkgo), previous gait speed, cognitive status, hospitalization, polypharmacy^a^, and previous number of falls (categorical). ^a^Stratified variables
