# Supplementary material for: Change in gait speed and fall risk among community-dwelling older adults with and without mild cognitive impairment: a retrospective cohort analysis
Source: BMC Geriatr. 2023 May 25;23:328. doi: 10.1186/s12877-023-03890-6 (PMC10214622; doi:10.1186/s12877-023-03890-6)
Supplement: Supplementary file 2 — Table S2. The association between change in gait speed and fall risk, competing risks analysis. (N=2755 observations=10424) [file 12877_2023_3890_MOESM2_ESM.docx]

**Table S2. The association between change in gait speed and fall risk, competing risks analysis. (N=2755 observations=10424)**

| Model | HR | 95% CI |
| --- | --- | --- |
| All Falls |  |  |
| **Unweighted**  Reference is no change (0.10 m/s faster to 0.10 m/s slower)  Faster  Slower | 0.97  1.11 | 0.87 to 1.09  1.00 to 1.23 |
| **Weighted^b^**  Reference is no change (0.10 m/s faster to 0.10 m/s slower)  Faster  Slower | 0.98  1.12 | 0.87 to 1.09  1.01 to 1.25 |
| Multiple Falls |  |  |
| **Unweighted**  Reference is no change (0.10 m/s faster to 0.10 m/s slower)  Faster  Slower | 1.05  1.40 | 0.85 to 1.29  1.14 to 1.72 |
| **Weighted^b^**  Reference is no change (0.10 m/s faster to 0.10 m/s slower)  Faster  Slower | 0.99  1.42 | 0.79 to 1.23  1.14 to 1.76 |

**Notes:** ^a^Stratified variables. All models adjusted for gender^a^, study site^a^, and treatment (Ginkgo), previous gait speed, cognitive status, polypharmacy^a^, and previous number of falls (categorical). ^b^Weights truncated to between >1% and <99% to remove negative values.
