# Supplementary figures and images for: Change in gait speed and fall risk among community-dwelling older adults with and without mild cognitive impairment: a retrospective cohort analysis
Source: BMC Geriatr. 2023 May 25;23:328. doi: 10.1186/s12877-023-03890-6 (PMC10214622; doi:10.1186/s12877-023-03890-6)

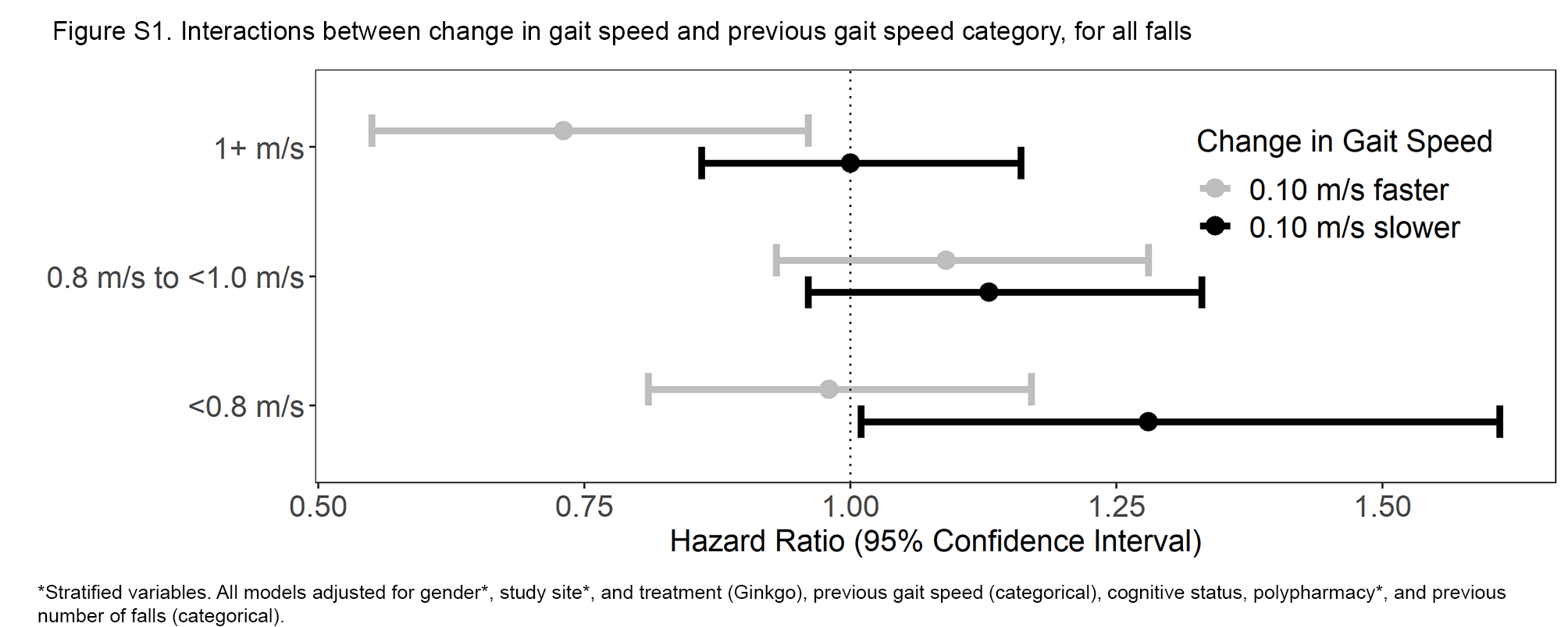

Supplement: Supplementary file 3 — Figure S1. Interactions between change in gait speed and previous gait speed category, for all falls [file 12877_2023_3890_MOESM3_ESM.png]

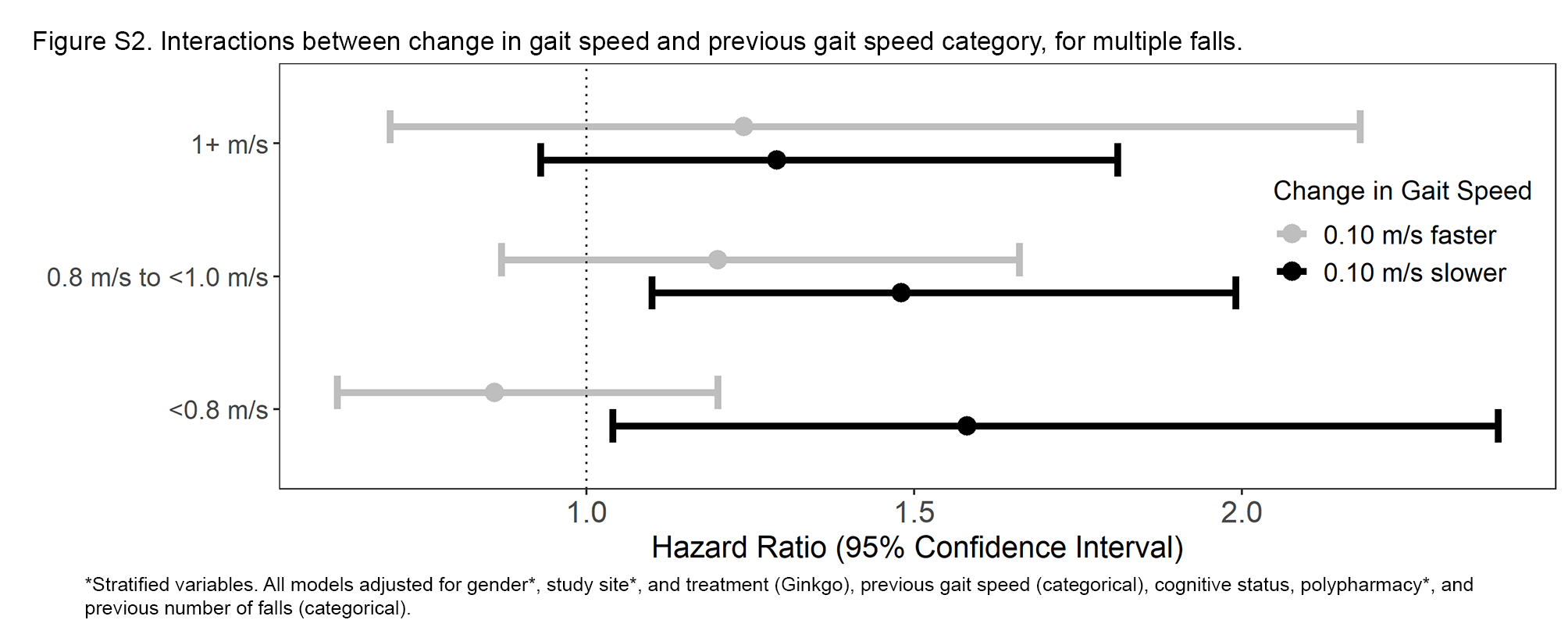

Supplement: Supplementary file 4 — Figure S2. Interactions between change in gait speed and previous gait speed category, for multiple falls [file 12877_2023_3890_MOESM4_ESM.png]

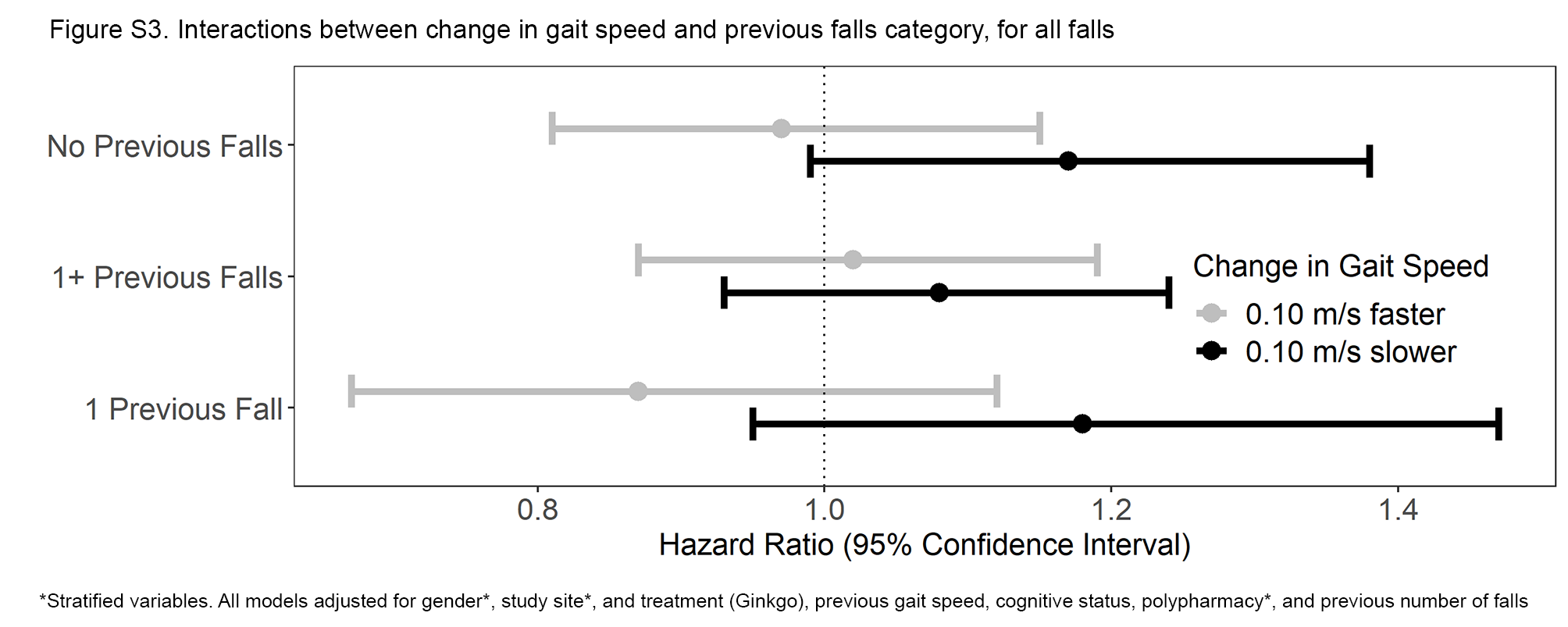

Supplement: Supplementary file 5 — Figure S3. Interactions between change in gait speed and previous falls category, for falls [file 12877_2023_3890_MOESM5_ESM.png]
